# Supplementary material for: Valproic Acid-Induced CCN1 Promotes Osteogenic Differentiation by Increasing CCN1 Protein Stability through HDAC1 Inhibition in Tonsil-Derived Mesenchymal Stem Cells
Source: Cells. 2022 Feb 3;11(3):534. doi: 10.3390/cells11030534 (PMC8834451; doi:10.3390/cells11030534)
Supplement: Supplementary file 1 [file cells-11-00534-s001.zip › cells-1533234-supplementary.pdf]

Supplementary Materials:

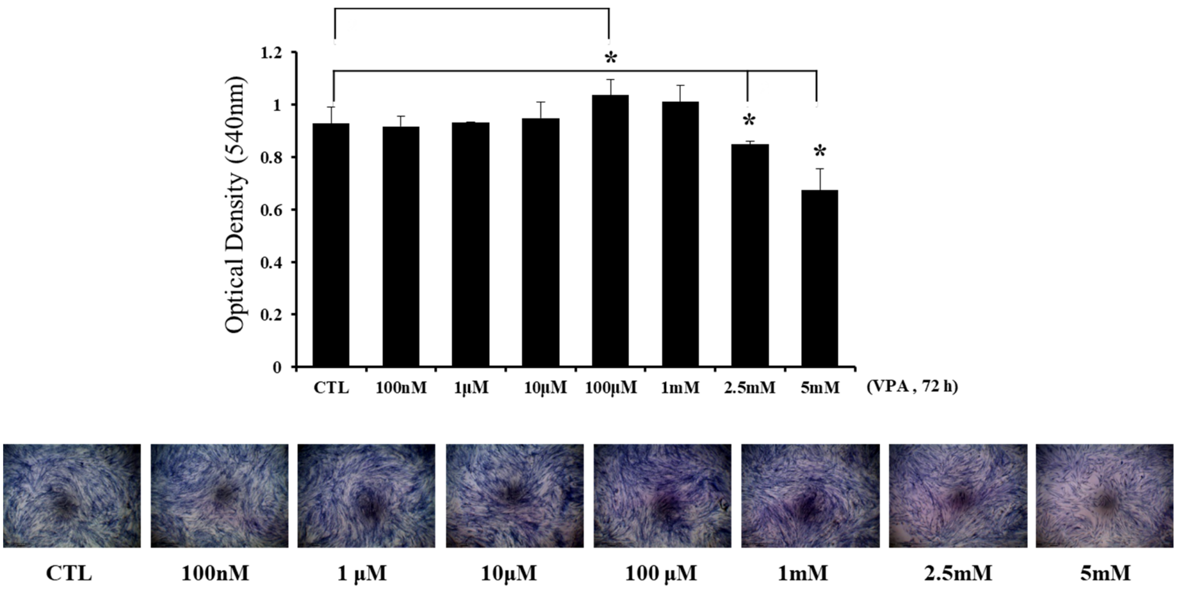

Figure S1. MTT assay to assess the effect of VPA at 0, 0.0001, 0.001, 0.01, 0.1, 1.0, 2.5 or 5.0 mM on the proliferation of TMSCs.

TMSCs (5.0×10^3 cells) were plated in 96 well plates and treated with 0, 0.0001, 0.001, 0.01, 0.1, 1.0, 2.5 or 5.0 mM of VPA for 72 h. After the VPA exposure, MTT (0.5mg/ml) assay solution was added and incubated for 3 h. The solution was removed, formazan crystal were dissolved in DMSO, and the optical density was measured at 540 nm using Synergy H1M (Biotek, Winooski, Vermont, USA). Statistical significance among different concentrations was denoted as *, $p < 0.05$, which were determined by one-way ANOVA followed by TUKEY'S analysis using GraphPad Prism software (GraphPad Software Inc. La Jolla, CA, USA). The results are representative of three independent experimental trials.

As shown in the Figure, 2.5 and 5 mM of VPA significantly decreased the proliferation of the TMSCs ($p < 0.05$). However, VPA significantly increased the proliferation at 100 μM ($p < 0.05$) but had no significant effect at concentrations less than 10 μM.

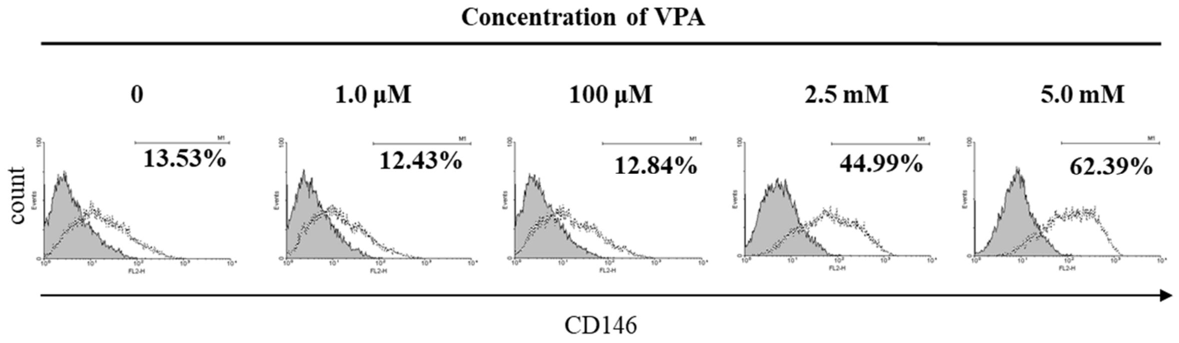

Figure S2. The effect of VPA at 0.001, 0.1, 2.5 and 5.0 mM on the surface CD146 expression of the TMSCs.

The TMSCs were exposed to 0.001, 0.1, 2.5 and 5.0 mM of VPA for 72 h. The TMSCs were detached using 1x TrypLE solution, and approximately 5.0×10^4 cells were incubated for 30 min at 4 °C with fluorescein isothiocyanate (FITC)-labeled antibodies against human CD146 (BD Biosciences) followed by its isotype controls (BD Biosciences). After washing the cells, the level of surface CD146 was analyzed using the FACScalibur flow cytometer (BD Biosciences). VPA at 0.001 and 0.1 μM did not affect the surface CD146 level, but VPA at 2.5 and 5 mM dramatically increased CD146 from 13.53 % to 44.99% and 62.39 %, respectively, compared to the control TMSCs.

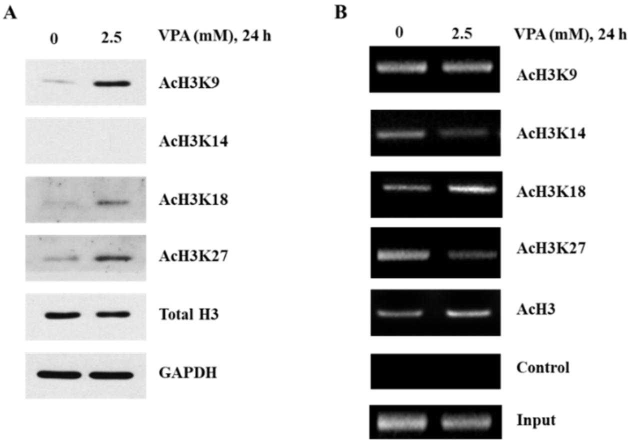

Figure S3. Effect of VPA on the acetylation at lysine residues of histone 3 (H3K9, H3K14, H3K18, H3K27).

(A) The acetylation level of histone 3 (H3) at Lysine 9, 14, 18 and 27 from the TMSCs (2.0 x 10^5 cells/mL) exposed to 0 and 2.5 mM of VPA for 24 h. Whole protein lysates were extracted using lysis buffer (20 mM Tris pH 7.5, 150 mM NaCl, 1 mM EDTA, 1 mM EGTA, 1 % Triton-X 100, 0.1 mM Na_3VO_4, 2 μg/ml leupeptin and 100 μg /ml PMSF). The protein concentrations were quantified using a BCA protein assay (Sigma-Aldrich). Equal quantities of protein (20 μg) were separated on 8 - 15 % SDS-PAGE, and they were transferred onto the nitrocellulose membrane. The protein level of total H3 and GAPDH was used as a reference to the H3 acetylation level.

Our earlier study found that the overexpression of HDAC1 dramatically decreased the CCN1 expression (Figure 7), suggesting that the inhibition of HDAC1 by VPA is crucial for inducing CCN1. We looked at some of the H3 acetylation sites, including H3K9, H3K14, H3K18 and H3K27, all of which are known to be important for regulating gene expression, and found that 2.5 mM of VPA exposure for 24 h increased the acetylation of H3 at lysine 9, 18 and 27. The results are representative of three independent experimental trials.

(B) A ChIP assay was performed using a ChIP assay kit (Millipore), according to the manufacturer's instructions and as described previously (Nelson et al. Nat Protoc 2006, 1(1): 179-185). Briefly, TMSCs treated with VPA were crosslinked in 1% formaldehyde and 0.1 M glycine and suspended in SDS lysis buffer (1% SDS, 10 mM EDTA and 50 mM Tris/HCl, pH8.1). The chromatin solution was sonicated, pre-cleared, and immunoprecipitated with 2 μg of the desired antibodies and protein A-agarose/Salmon Sperm DNA beads. Antibodies against histone H3 acetylated at lysine 9 (AcH3K9), AcH3K14 AcH3K18 and AcH3K27 were purchased from Cell Signaling Technology (Danvers, MA, USA). Mock samples were prepared by the immunoprecipitation procedure without antibody. Input (total chromatin extract), mock (Control) and ChIP samples were recovered and used for PCR analyses. PCR amplification of the CCN1 promoter region was conducted in a total volume of 20 μL using Platinum PCR SuperMix (Invitrogen). Primers used for ChIP assay in the experiment were as follows: *CCN1*, 5'- CAG TCC AGG CAA AGT TCT GA -3' (forward) and 5'- GTG TGT GTG TGT CCC AGA CC -3' (reverse). The results are representative of three independent experimental trials. As shown in Figure S3, 2.5 mM of VPA decreased the binding of AcH3K14 and K27 at the CCN1 promoter, but increased the binding of AcH3K18, suggesting that AcH3K18 could be the primary acetylation site that increases the promoter activity of CCN1.

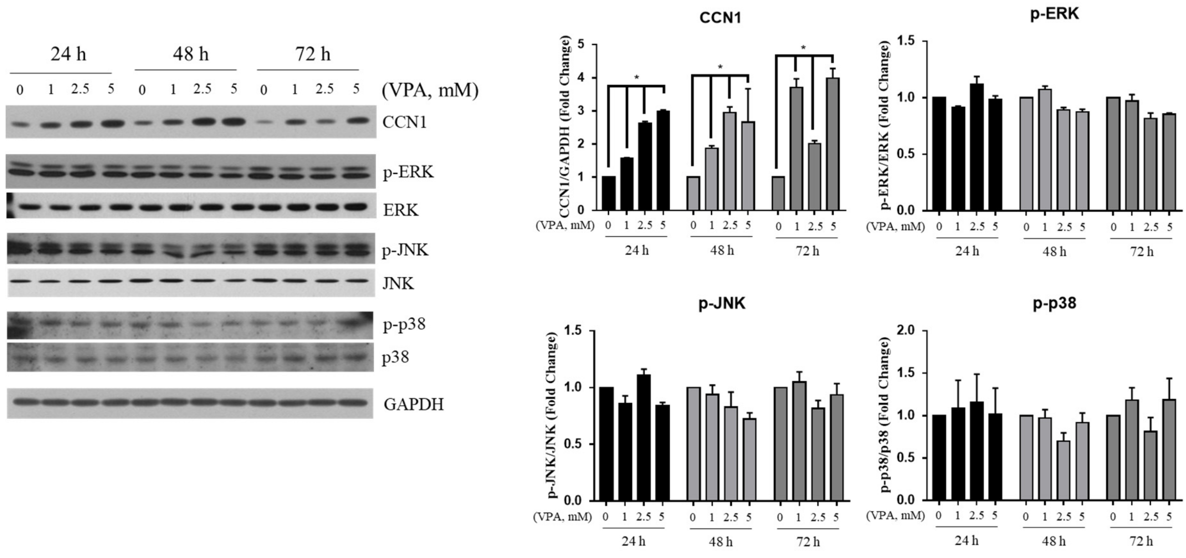

Figure S4. The protein level of CCN1 as well as the phosphorylated forms of ERK, JNK and p38 after 1.0, 2.5 and 5.0 mM of VPA exposure for 24, 48 and 72 h.

The TMSCs were seeded at 2.0 x 10^5 cells/mL of density followed by VPA exposure at 0, 1, 2.5 and 5 mM for 24, 48 and 72 h. Whole protein lysates were extracted using lysis buffer (20 mM Tris pH 7.5, 150 mM NaCl, 1 mM EDTA, 1 mM EGTA, 1 % Triton-X 100, 0.1 mM Na_3VO_4, 2 μg/ml leupeptin and 100 μg /ml PMSF). The protein concentrations were quantified using a BCA protein assay (Sigma-Aldrich). Equal quantities of protein (20 μg) were separated on 8 - 15 % SDS-PAGE, and they were transferred onto the nitrocellulose membrane. The blots were then probed with a primary antibody against CCN1, ERK, phosphorylated ERK (p-ERK), JNK, p-JNK, p38 and p-p38 followed by their corresponding secondary antibodies. GAPDH was used as a reference protein. The protein blots were developed using enhanced chemiluminescence reagents (Amersham, Buckinghamshire, UK). Statistical significance among different concentrations was denoted as *, $p < 0.05$, which were determined by one-way ANOVA followed by TUKEY'S analysis using GraphPad Prism software (GraphPad Software Inc. La Jolla, CA, USA). The results are representative of three independent experimental trials.

It was shown that various stress conditions, such as mechanical strain, induce CC1 through p38 stress-activated protein kinase (SAPK). Therefore, we assessed the phosphorylation of ERK, JNK and p38 to determine if the VPA induces CCN1 through p38 SAPK. As already reported in Figures 6A and B of the original manuscript, the CCN1 level was significantly increased at 2.5 and 5 mM of VPA for 24 and 48 h, and its induction was diminished at 72 h of exposure. However, VPA did not affect p-ERK, p-JNK and p-p38 regardless of the CCN1 protein level.
